# Supplementary material for: geneBasis: an iterative approach for unsupervised selection of targeted gene panels from scRNA-seq
Source: Genome Biol. 2021 Dec 6;22:333. doi: 10.1186/s13059-021-02548-z (PMC8650258; doi:10.1186/s13059-021-02548-z)
Supplement: Supplementary file 1 — Additional file 1: Fig. S1. Schematic visualisation for cell neighbourhood preservation score workflow. Fig. S2. Systematic assessment of the ‘completeness’ of the generated gene panels. Fig. S3. SCMER introduces redundancy in the selections when selecting a small number of genes. Fig. S4. geneBasis is robust to initial selections and quickly finds missing sources of variation. Fig. S5. geneBasis accounts for batch effects even with highly unbalanced celltype composition. Fig. S6. Detailed analysis of the gene selection for mouse embryogenesis. Fig. S7. Detailed analysis of the selection for spleen and pancreas . Panels A-D correspond to spleen; panels E-G correspond to pancreas. Fig. S8. geneBasis selects genes that recover biological heterogeneity in seqFISH datasets. Fig. S9. Performing PCA for mapping manifolds with selections does not change cell neighborhood preservation score or gene prediction score. Fig. S10. Minkowski distance of order p=3 provides the resolution to select rare markers and genes expressed in a low fraction of cells. [file 13059_2021_2548_MOESM1_ESM.pdf]

Fig. S1

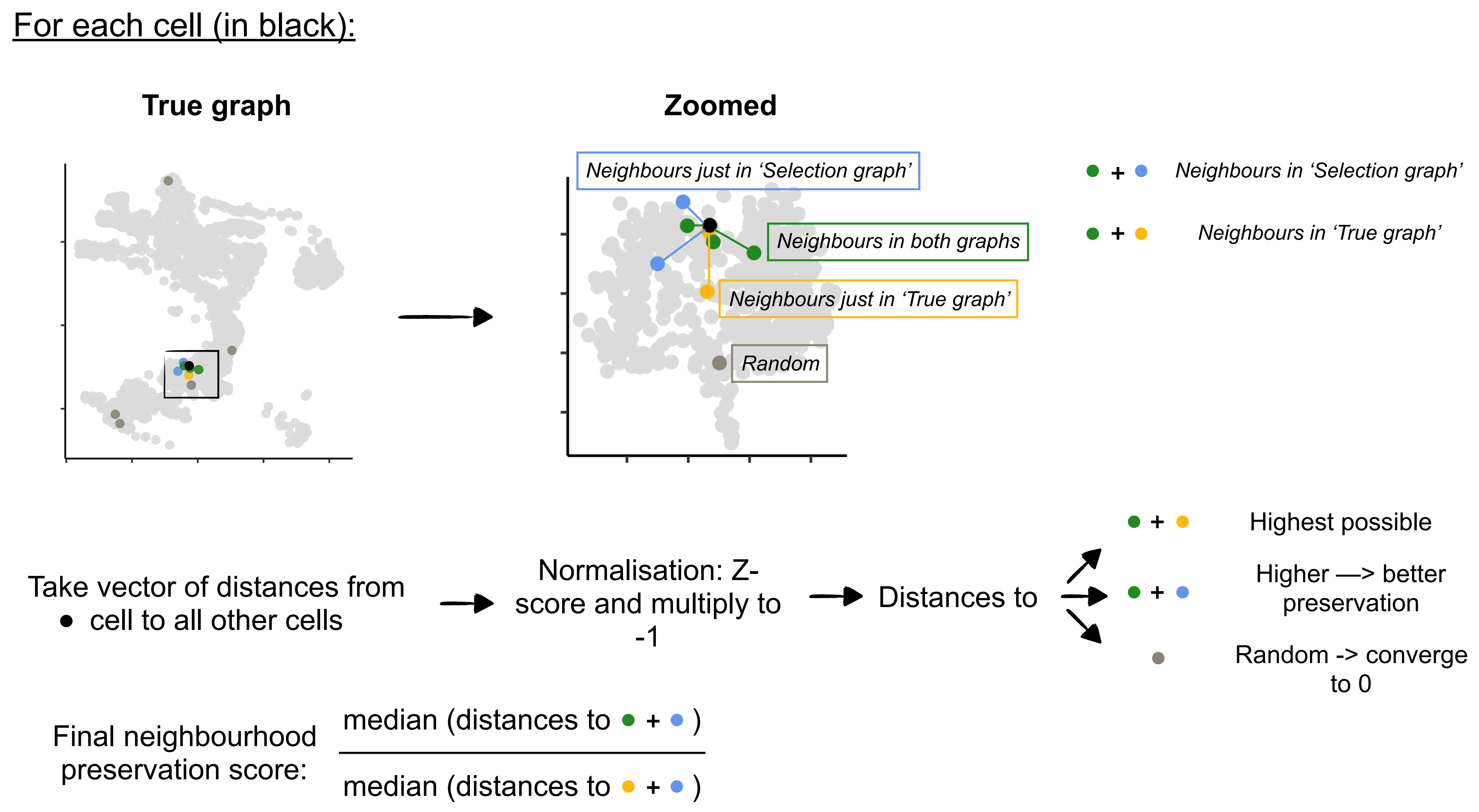

Fig. S1. Schematic visualisation for cell neighbourhood preservation score workflow. For each cell (in black) we detect neighbours from the True graph and from the Selection graph, and use the ratio of normalised distances as the final score.

Fig. S2

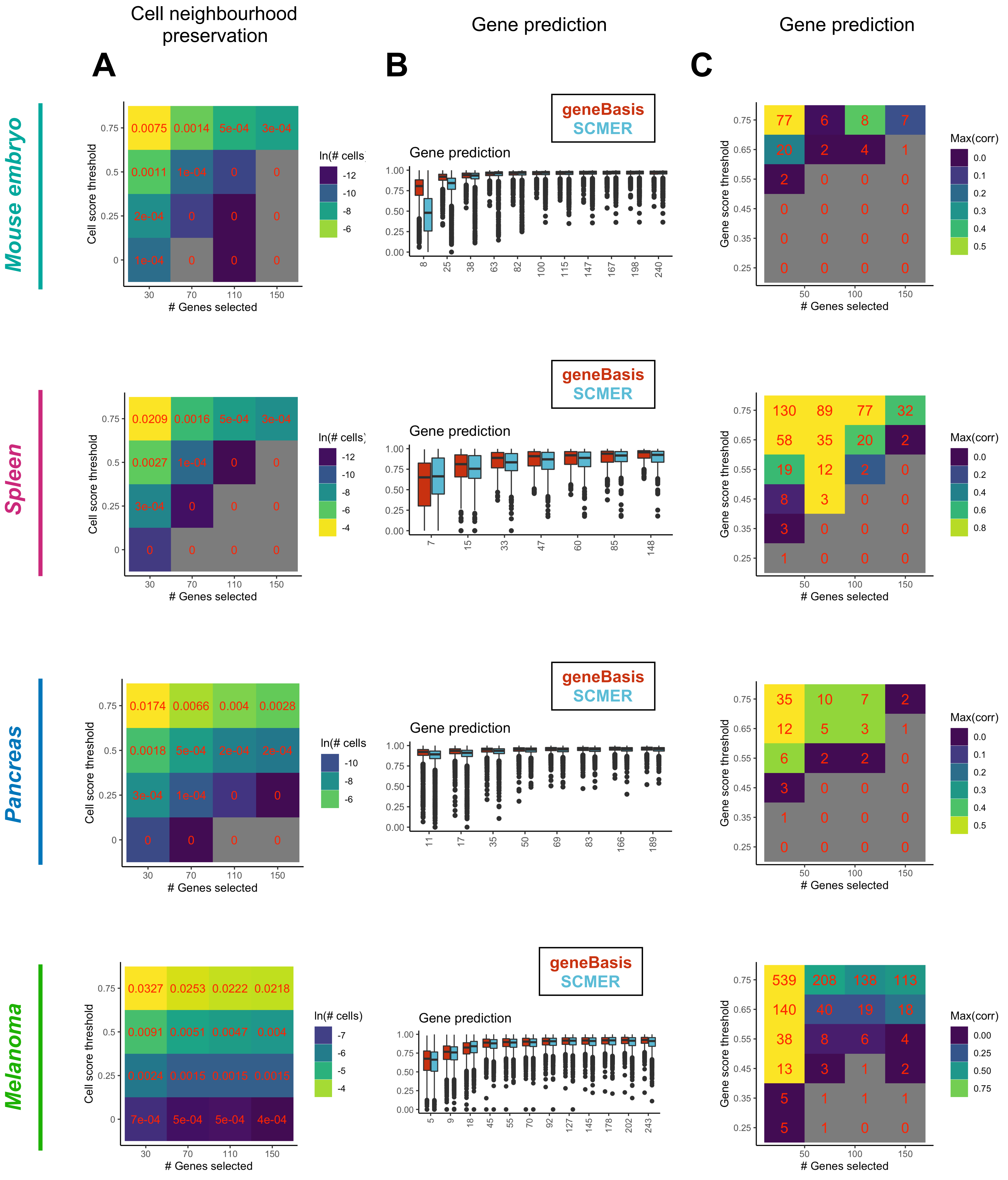
Cell neighbourhood preservation
Gene prediction
Gene prediction
A
B
C
Mouse embryo
Spleen
Pancreas
Melanoma
geneBasis
SCMER
Gene prediction
Cell score threshold
Genes selected
Gene score threshold
ln(# cells)
Max(corr)

Fig. S2. Systematic assessment of the ‘completeness’ of the generated gene panels. We provide several semi-orthogonal metrics to evaluate how complete the current panel is. Analysis is performed for each dataset (in rows).

(A) Heatmap represents the fraction of cells that have low neighbourhood preservation score - as a function of number of genes (X-axis) and threshold for neighbourhood preservation score (Y-axis). Colours are log-scaled to facilitate visualization. Numbers inside correspond to actual values.

(B) Distribution of gene prediction score as a function of the gene panel size. Colours correspond to different methods.

(C) Heatmap represents statistics regarding gene prediction score - as a function of number of genes (X-axis) and threshold for gene prediction score (Y-axis). Numbers inside correspond to the number of genes that show a gene prediction score lower than the corresponding threshold. Colours correspond to maximum across correlations for all pairwise comparisons between genes with low gene prediction scores. Colours are log-scaled to facilitate visualisation.

Fig. S3

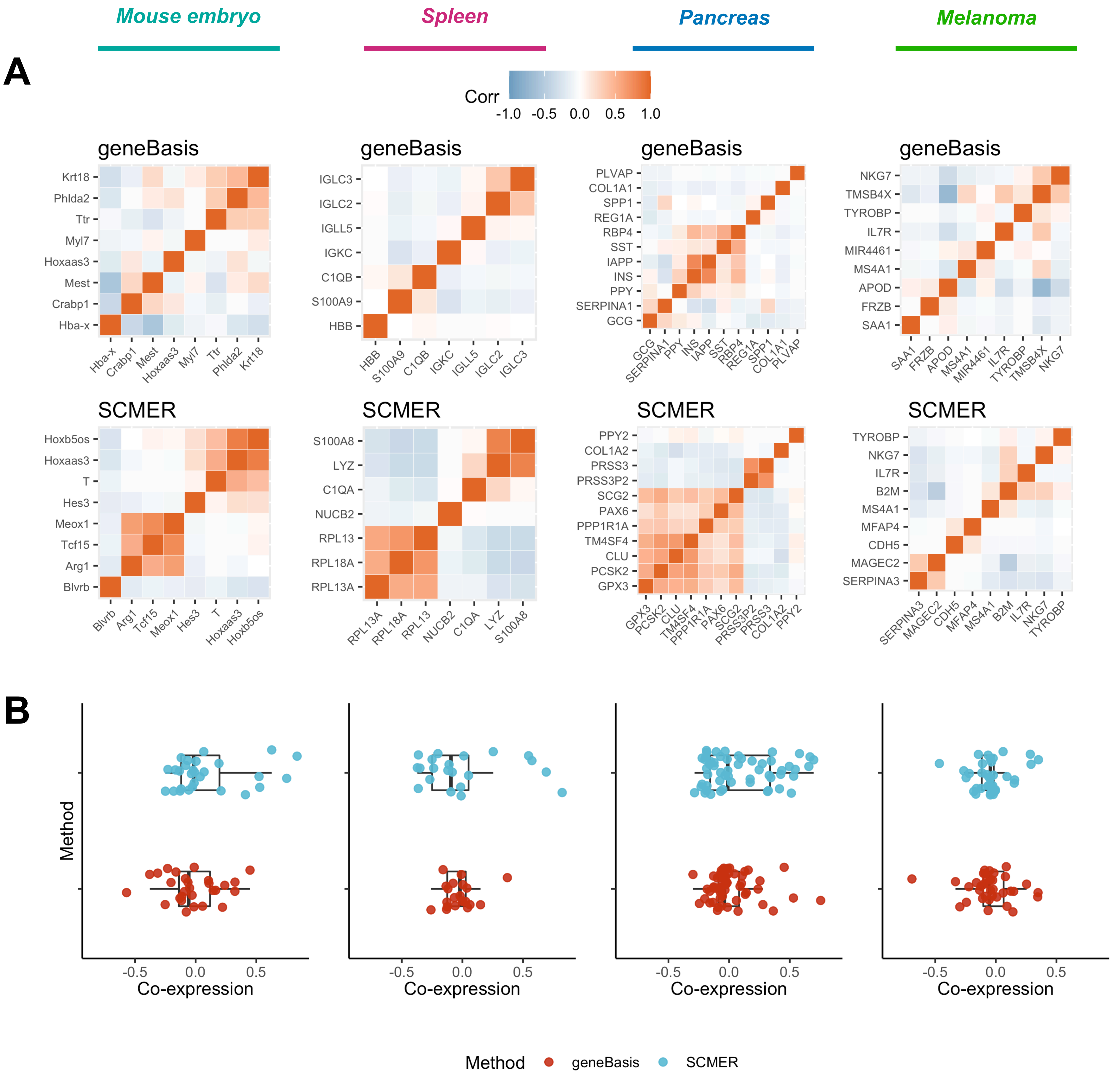

Fig. S3. SCMER introduces redundancy in the selections when selecting a small number of genes.

(A) Heatmaps showing the co-expression (Spearman correlation) between genes selected by geneBasis and SCMER. The overall degree of co-expression is lower for geneBasis compared to SCMER, and this is consistent for all benchmarked datasets.

(B) Boxplots represent pairwise correlation in log-normalized expression values (Y-axis) for the first genes selected for geneBasis (red) and SCMER (light blue).

Fig. S4

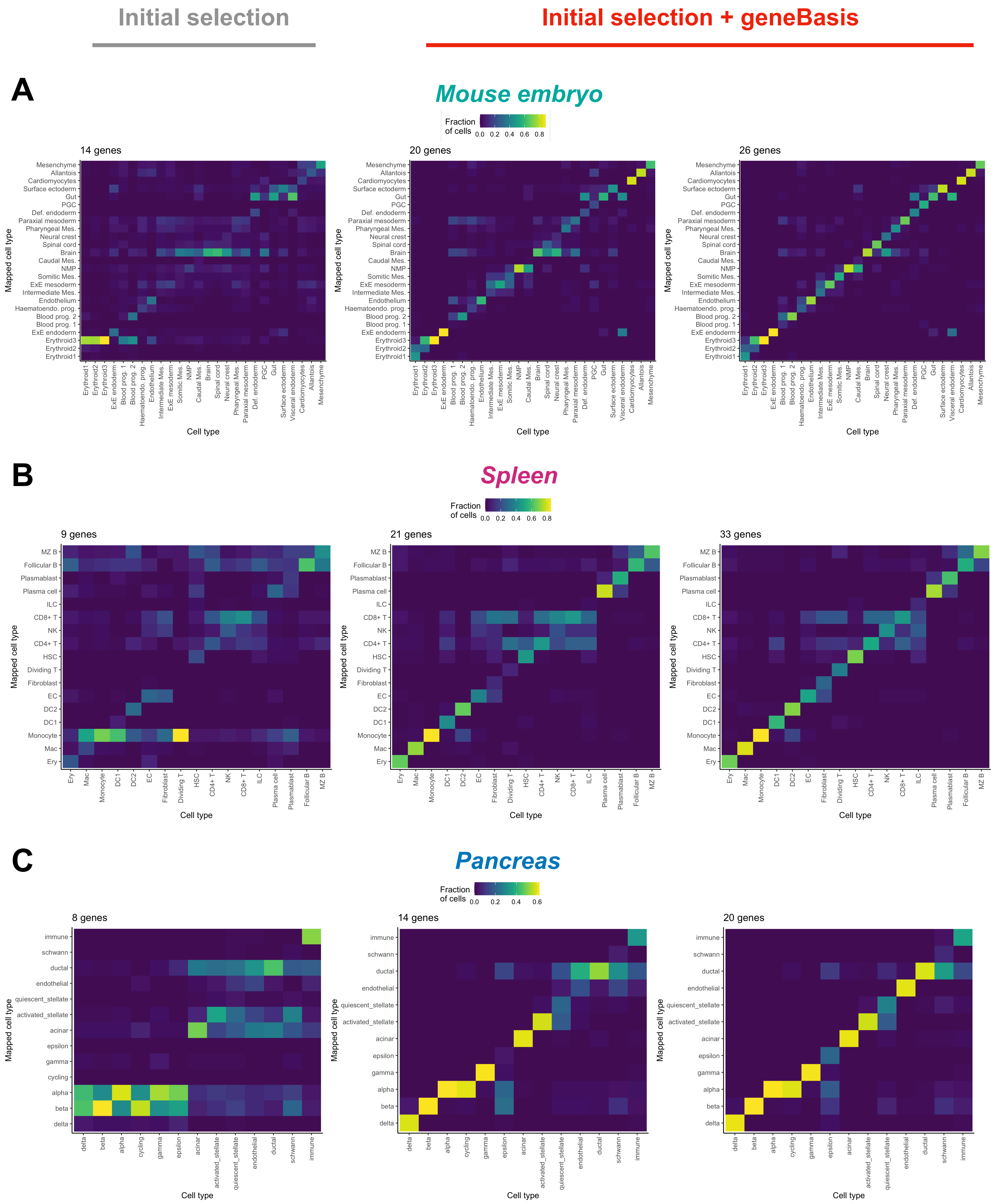

Initial selection
Initial selection + geneBasis
A
Mouse embryo
Fraction of cells
0.0 0.2 0.4 0.6 0.8
14 genes
20 genes
26 genes
Mesenchyme
Allantois
Cardiomyocytes
Surface ectoderm
Gut
PGC
Def. endoderm
Paraxial mesoderm
Pharyngeal Mes.
Neural crest
Spinal cord
Brain
Caudal Mes.
NMP
Somitic Mes.
ExE mesoderm
Intermediate Mes.
Endothelium
Haematoendo. prog.
Blood prog. 2
Blood prog. 1
ExE endoderm
Erythroid3
Erythroid2
Erythroid1
Visceral endoderm
Mapped cell type
Cell type
B
Spleen
Fraction of cells
0.0 0.2 0.4 0.6 0.8
9 genes
21 genes
33 genes
MZ B
Follicular B
Plasmablast
Plasma cell
ILC
CD8+ T
NK
CD4+ T
HSC
Dividing T
Fibroblast
EC
DC2
DC1
Monocyte
Mac
Ery
Mapped cell type
Cell type
C
Pancreas
Fraction of cells
0.0 0.2 0.4 0.6
8 genes
14 genes
20 genes
immune
schwann
ductal
endothelial
quiescent_stellate
activated_stellate
acinar
epsilon
gamma
cycling
alpha
beta
delta
Mapped cell type
Cell type

Fig. S4. geneBasis is robust to initial selections and quickly finds missing sources of variation.

(A) Mouse embryo. Celltype confusion matrices for the initial semi-random selection (left), for the updated selection with 6 additional genes (middle) and updated selection with 12 additional genes (right).

(B) Spleen. Celltype confusion matrices for the initial semi-random selection (left), for the updated selection with 12 additional genes (middle) and updated selection with 24 additional genes (right).

(C) Pancreas. Celltype confusion matrices for the initial semi-random selection (left), for the updated selection with 6 additional genes (middle) and updated selection with 12 additional genes (right).

Fig S5

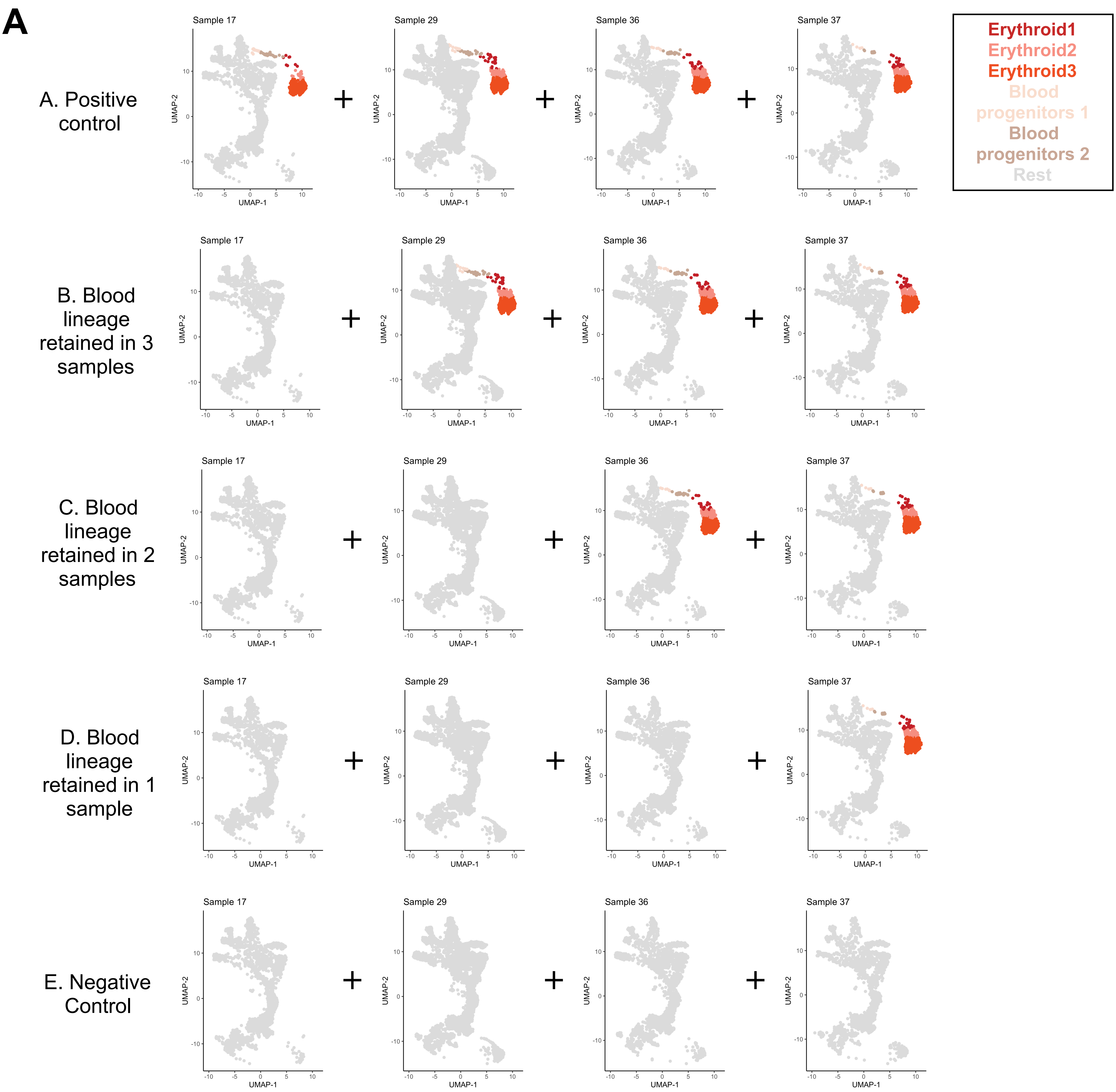

B

	A	B	C	D	E
gene Basis	+	+	+	+	-
SCMER	+	+	+	-	-

\+ At least one blood marker is selected

\- No blood markers are selected

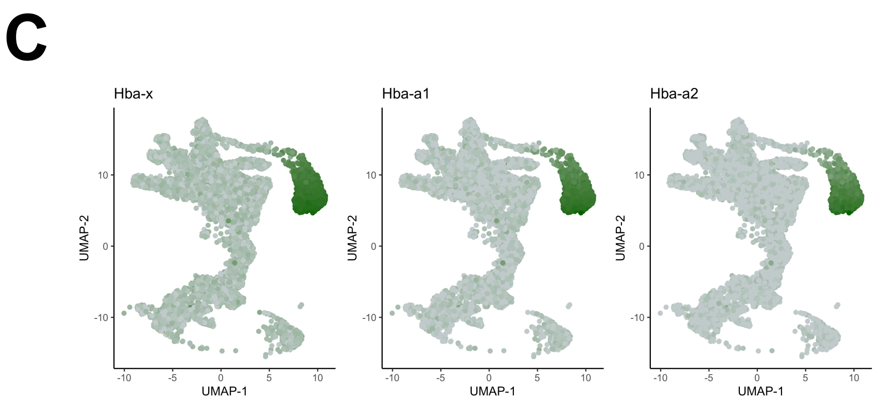

Fig. S5. geneBasis accounts for batch effects even with highly unbalanced celltype composition.

(A) Overview of the *in silico* experiment. Gene search was performed for 5 datasets (in rows), and inclusion of blood lineage for each sample (i.e. batch) is specified in columns.

(B) Table represents whether blood markers were selected for each dataset and method.

(C) UMAPs for mouse embryogenesis, coloured by expression of the selected blood marker genes.

Fig. S6

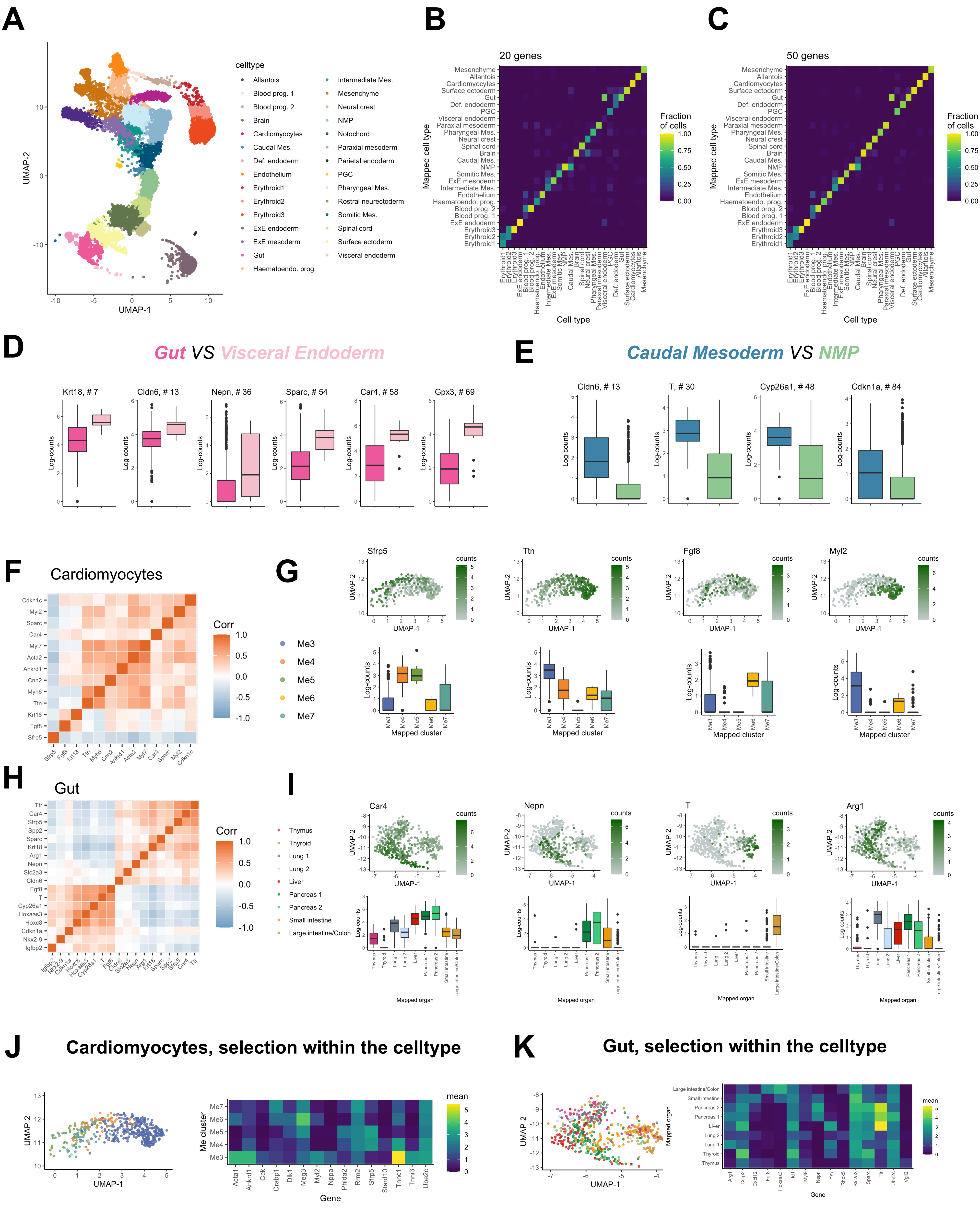
A
B
C
celltype
Allantois
Blood prog. 1
Blood prog. 2
Brain
Cardiomyocytes
Caudal Mes.
Def. endoderm
Endothelium
Erythroid1
Erythroid2
Erythroid3
ExE endoderm
ExE mesoderm
Gut
Haematoendo. prog.
Intermediate Mes.
Mesenchyme
Neural crest
NMP
Notochord
Paraxial mesoderm
Parietal endoderm
PGC
Pharyngeal Mes.
Rostral neurectoderm
Somitic Mes.
Spinal cord
Surface ectoderm
Visceral endoderm
UMAP-1
UMAP-2
20 genes
50 genes
Mapped cell type
Cell type
Fraction of cells
D
Gut VS Visceral Endoderm
Krt18, # 7
Cldn6, # 13
Nepn, # 36
Sparc, # 54
Car4, # 58
Gpx3, # 69
Log-counts
E
Caudal Mesoderm VS NMP
Cldn6, # 13
T, # 30
Cyp26a1, # 48
Cdkn1a, # 84
F
Cardiomyocytes
Corr
G
Me3
Me4
Me5
Me6
Me7
Sfrp5
Ttn
Fgf8
Myl2
counts
Mapped cluster
H
Gut
I
Thymus
Thyroid
Lung 1
Lung 2
Liver
Pancreas 1
Pancreas 2
Small intestine
Large intestine/Colon
Car4
Nepn
T
Arg1
Mapped organ
J
Cardiomyocytes, selection within the celltype
Me cluster
Gene
mean
K
Gut, selection within the celltype
Mapped organ
Gene
mean

Fig. S6. Detailed analysis of the gene selection for mouse embryogenesis.

(A) UMAP representation coloured by celltypes. Note that it duplicates part of Figure 2C and is introduced here solely to facilitate interpretation of the data.

(B) Celltype confusion matrix for the first 20 selected genes.

(C) Celltype confusion matrix for the first 50 selected genes.

(D) Box Plots representing bulk log-normalised expression across celltypes for genes that are differentially expressed between Visceral endoderm and Gut.

(E) Box Plots representing bulk log-normalised expression across celltypes for genes that are differentially expressed between Caudal mesoderm and NMP.

(F) Co-expression (within Cardiomyocytes) of genes prioritised by geneBasis and differentially expressed in Cardiomyocytes.

(G) For manually selected genes denoted as relevant to inter-cardiomyocytes heterogeneity: UMAP representation of Cardiomyocytes coloured by expression and box plots representing bulk log-normalised expression across mapped cardiac clusters.

(H) Co-expression (within Gut cells) of genes prioritised by geneBasis and differentially expressed in Gut.

(I) For manually selected genes denoted as relevant to inter-gut heterogeneity: UMAP representation of Gut cells colored by expression and box plots representing bulk log-normalised expression across mapped cardiac clusters.

(J) Left panel: UMAP representation of Cardiomyocytes, coloured by mapped cardiac clusters, when integration between datasets was performed using the first 15 genes prioritised by geneBasis if performed directly on Cardiomyocytes. Right panel: Heatmap representing average expression for assigned (using mapping with all genes) cardiac cluster (Y-axis) for first 15 genes prioritised by geneBasis if performed directly on Cardiomyocytes.

(K) Left panel: UMAP representation of Gut cells, coloured by mapped cardiac clusters, when integration between datasets was performed using first 15 genes prioritised by geneBasis if performed directly on Gut cells. Right panel: Heatmap representing average expression for assigned (using mapping with all genes) gut suborgan (Y-axis) for first 15 genes prioritised by geneBasis if performed directly on Gut cells.

Fig. S7

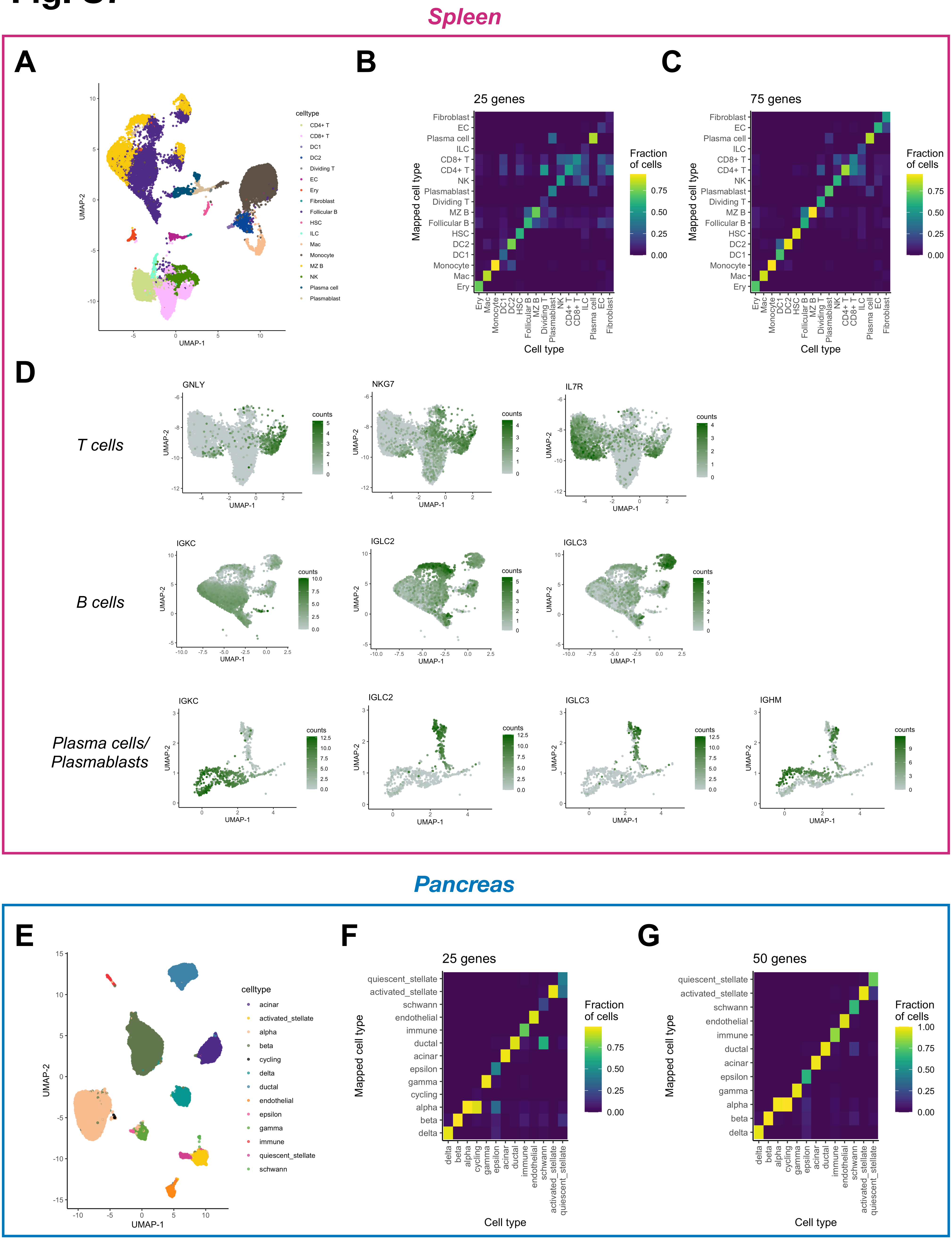

Spleen
A
celltype
CD4+ T
CD8+ T
DC1
DC2
Dividing T
EC
Ery
Fibroblast
Follicular B
HSC
ILC
Mac
Monocyte
MZ B
NK
Plasma cell
Plasmablast
UMAP-1
UMAP-2
B
25 genes
C
75 genes
Mapped cell type
Cell type
Fraction of cells
Fibroblast
EC
Plasma cell
ILC
CD8+ T
CD4+ T
NK
Plasmablast
Dividing T
MZ B
Follicular B
HSC
DC2
DC1
Monocyte
Mac
Ery
D
T cells
GNLY
NKG7
IL7R
counts
B cells
IGKC
IGLC2
IGLC3
Plasma cells/ Plasmablasts
IGHM
Pancreas
E
celltype
acinar
activated_stellate
alpha
beta
cycling
delta
ductal
endothelial
epsilon
gamma
immune
quiescent_stellate
schwann
F
25 genes
G
50 genes
Mapped cell type
Cell type
Fraction of cells

Fig. S7. Detailed analysis of the selection for spleen and pancreas . Panels A-D correspond to spleen; panels E-G correspond to pancreas.

(A) UMAP representation coloured by celltypes. Note that it duplicates part of Figure 2C and is introduced here solely to facilitate interpretation of the data.

(B) Celltype confusion matrix for 25 selected genes.

(C) Celltype confusion matrix for the first 75 selected genes.

(D) UMAP representation of T cells (Upper panel), B cells (Middle panel) and Plasma cells and Plamblasts (Lower panel), colored by genes relevant for inter-celltype variability within corresponding celltypes.

(E) UMAP representation colored by celltypes. Note that it duplicates part of Figure 2C and is introduced here solely to facilitate interpretation of the data.

(F) Celltype confusion matrix for 25 selected genes.

(G) Celltype confusion matrix for 50 selected genes.

Fig. S8

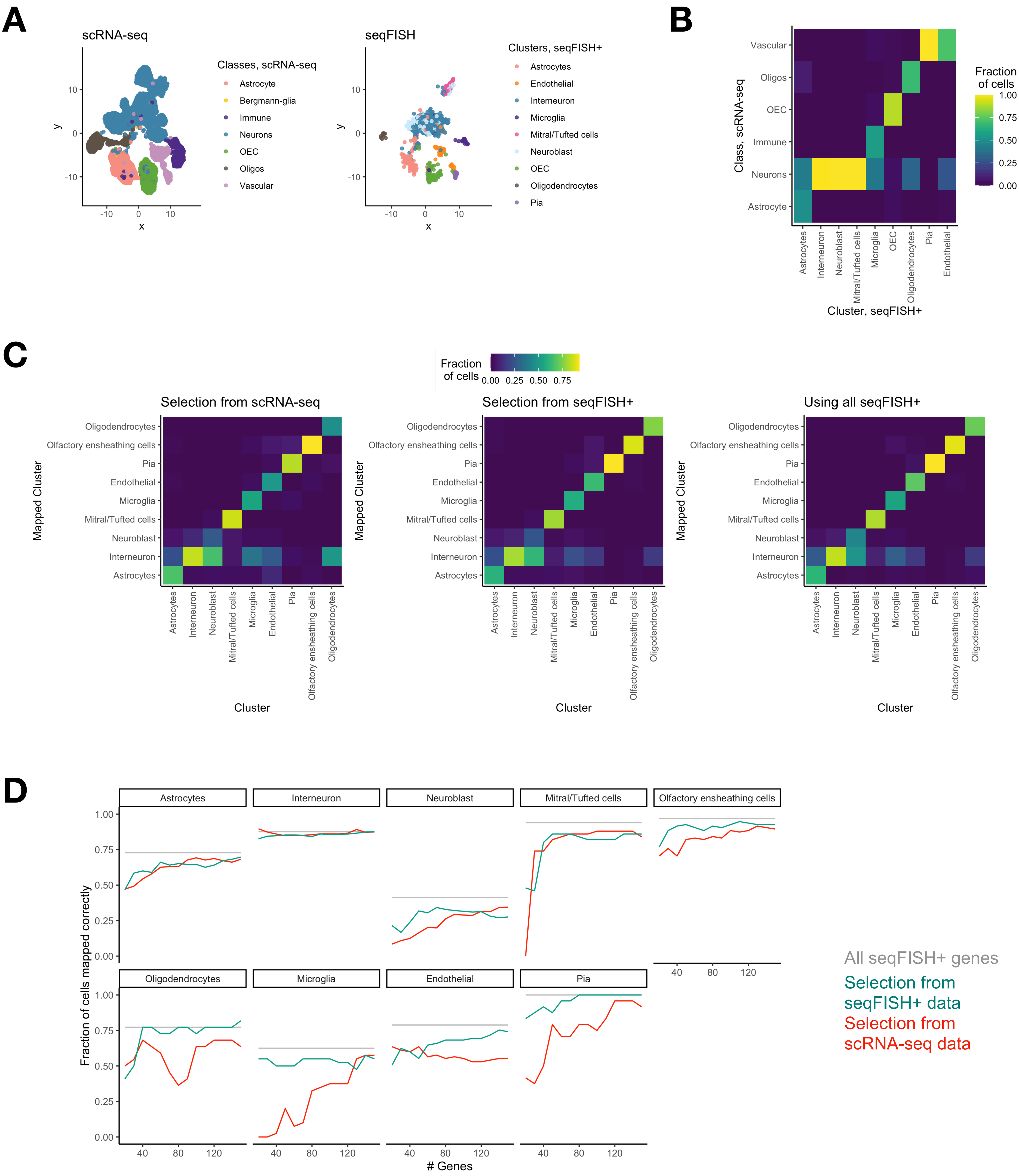
A
scRNA-seq
seqFISH
Classes, scRNA-seq
Astrocyte
Bergmann-glia
Immune
Neurons
OEC
Oligos
Vascular
Clusters, seqFISH+
Astrocytes
Endothelial
Interneuron
Microglia
Mitral/Tufted cells
Neuroblast
OEC
Oligodendrocytes
Pia
B
Class, scRNA-seq
Cluster, seqFISH+
Fraction of cells
C
Selection from scRNA-seq
Selection from seqFISH+
Using all seqFISH+
Mapped Cluster
Cluster
D
Astrocytes
Interneuron
Neuroblast
Mitral/Tufted cells
Olfactory ensheathing cells
Oligodendrocytes
Microglia
Endothelial
Pia
Fraction of cells mapped correctly
Genes
All seqFISH+ genes
Selection from seqFISH+ data
Selection from scRNA-seq data

Fig. S8. geneBasis selects genes that recover biological heterogeneity in seqFISH datasets.

(A) UMAP plots representing joint embedding for matched scRNA-seq (left) and seqFISH+ (right) of an olfactory bulb of a mouse. Colours correspond to respectively annotated cell types/clusters.

(B) Heatmap representing confusion matrix for seqFISH+ cells between cluster originally assigned in seqFISH+ (X-axis) and mapped Class from scRNA-seq (Y-axis).

(C) Heatmaps representing cell type mapping accuracy (for seqFISH+ data) for the selection of 150 genes derived from scRNA-seq (left); selection of 150 genes derived from seqFISH+ (center); all 10000 genes probed in seqFISH+.

(D) Cell type mapping accuracy as a function of the number of genes in the panel. Each facet corresponds to the cluster from seqFISH+, colours correspond to the origins of the gene panel.

Fig. S9

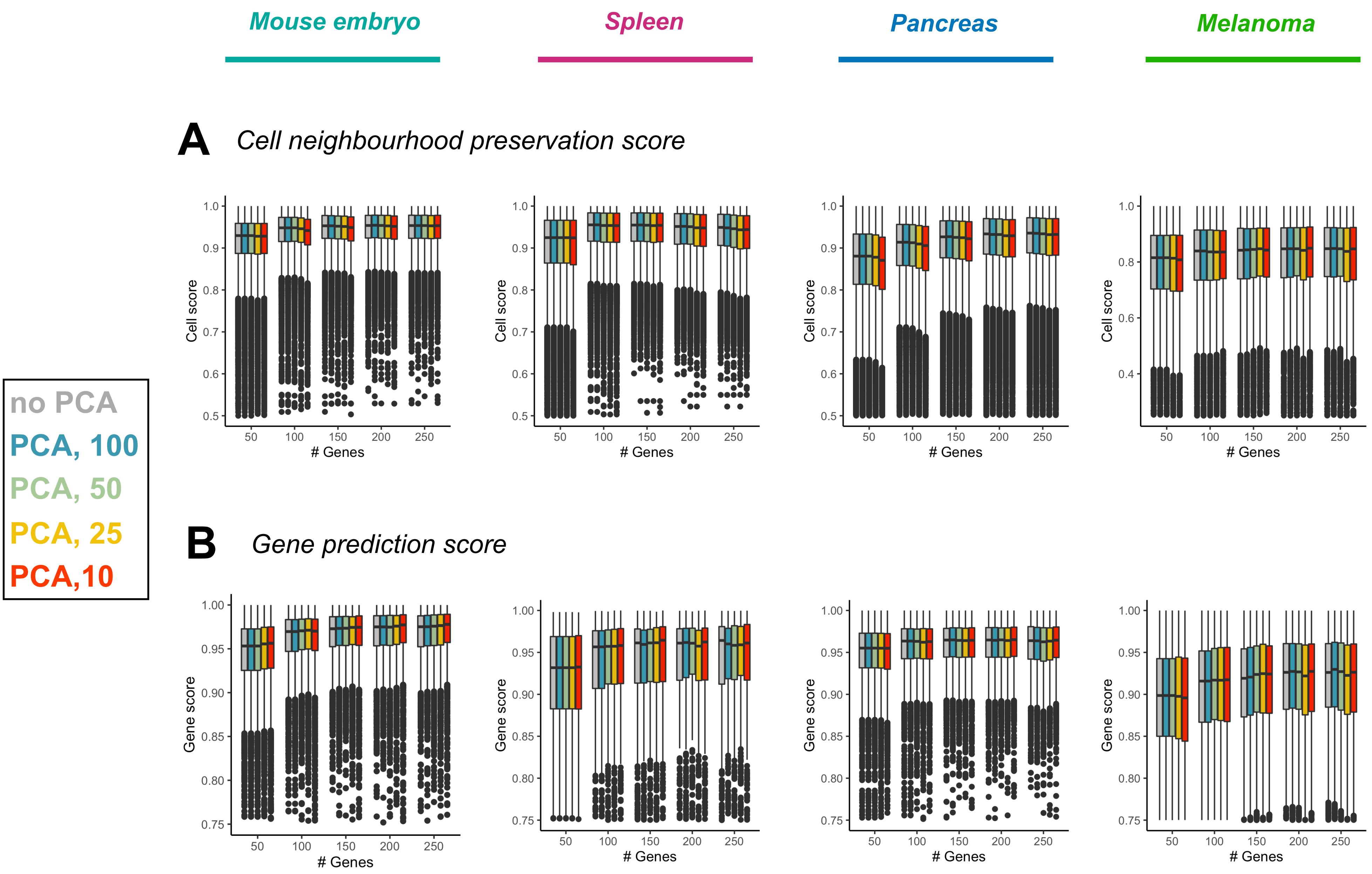

Fig. S9. Performing PCA for mapping manifolds with selections does not change cell neighborhood preservation score or gene prediction score.

(A) Box plots representing overall cell neighbourhood preservation score distribution as a function of the number of genes in the selections (X-axis) and optional PCA step with different number of components being selected + default no PCA (in colour).

(B) Box plots representing overall gene prediction score distribution as a function of number of genes in the selections (X-axis) and optional PCA step with different number of components being selected + default no PCA (in colour).

Fig. S10

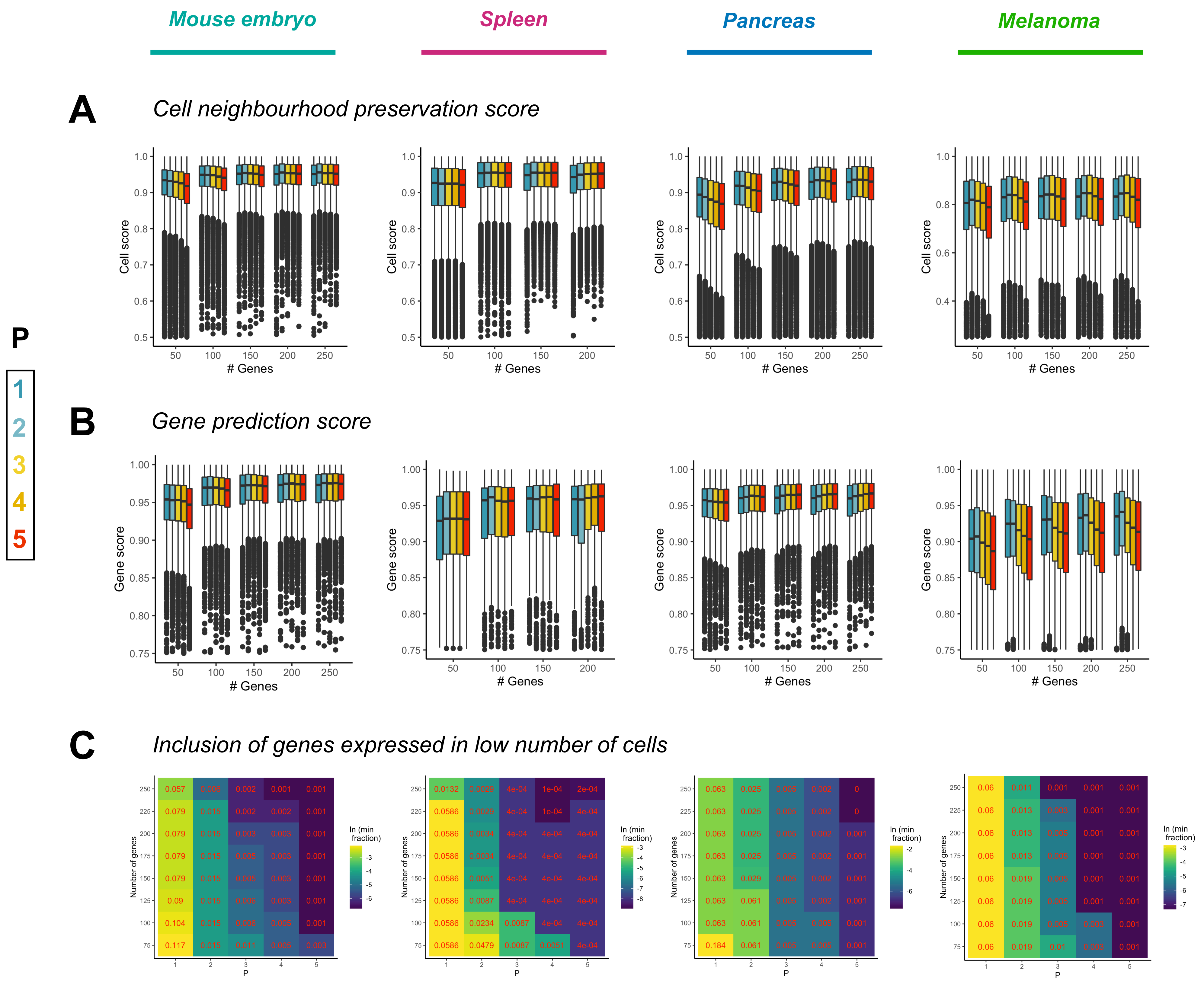

Fig. S10. Minkowski distance of order p=3 provides the resolution to select rare markers and genes expressed in a low fraction of cells. Each column corresponds to a single dataset.

(A) Box plots representing overall cell neighborhood preservation score distribution as a function of number of genes in the selections (X-axis) and different orders of Minkowski distance (p) used for the algorithm (in colour).

(B) Box plots representing overall gene prediction score distribution as a function of number of genes in the selections (X-axis) and p (orders of Minkowski distance) used for the algorithm (in colour).

(C) Heatmaps representing the ability of a selection with the given order of Minkowski distance to prioritise lowly expressed genes. X-axis corresponds to the choice of p, Y-axis corresponds to number of genes in the selection, and both numbers (absolute scale) and colours (log scale) correspond to the minimum of expression levels (in the context fraction of cells with non-zero counts) across selected genes.
