# Supplementary material for: geneBasis: an iterative approach for unsupervised selection of targeted gene panels from scRNA-seq
Source: Genome Biol. 2021 Dec 6;22:333. doi: 10.1186/s13059-021-02548-z (PMC8650258; doi:10.1186/s13059-021-02548-z)
Supplement: Supplementary file 5 — Additional file 5: Supplementary Note 2. Analysis of PBMC dataset. [file 13059_2021_2548_MOESM5_ESM.pdf]

Supplementary Note 2.

geneBasis captures signals associated with cell state changes in activated lymphocytes and monocytes.

To evaluate whether we can detect transcriptional changes upon lymphocyte and monocyte activation we applied geneBasis to the CITE-seq dataset profiling PBMCs from healthy donors that were treated with anti-CD3/CD28 and LPS [1]. Cell types were assigned using marker cell surface proteins, and shifts in transcriptional programs upon lymphocytes and monocytes activation were characterized within individual cell types. Overall, anti-CD3/CD28 treatment activated all lymphocytes, with some genes being ubiquitously altered in their expression whereas others showed cell type specific behaviour. In contrast, LPS treatment specifically altered the transcriptional state of the monocytes, resulting in a heterogeneous response comprised of an early activation and later pro-inflammatory states. To specifically select for genes associated with cell state changes, we performed the panel selections within each cell type separately (Methods).

First we analyzed cell type specific cell state changes for the lymphocyte activation dataset (Methods). To do so, we combined baseline cells (no treatment) together with anti-CD3/CD28 cells and applied geneBasis to select the top 25 genes for each lymphocyte related cell type (T cells, B cells and NK). Many of the genes selected in each set of 25 were differentially expressed between baseline and activated cells (**Additional file 5: Supplementary Note 2, Fig. 1A,B**), with some genes being consistently detected across multiple cell types. For example, ACTB, TMSB10 and NPM1 are slightly up-regulated across lymphocytes suggestive of cellular proliferation and movement. On the other hand, some genes were more cell type specific such as a proteasome activity (PSME2) in B cells. Importantly, we identified multiple signals of activation described in the original publication. Among others, we detected interferons and interferon-inducible genes (ISG15, IFNG); chemokines XCL1 and XCL2 in CD8+ cells; cytotoxic molecules GZMB and GNLY in CD8+ and NK cells; and human leukocyte antigens (HLA-B, HLA-C).

Next we analyzed monocyte specific cell state changes under LPS treatment. Out of the top 25 genes selected by geneBasis, 20 were up-regulated in activated monocytes, supporting the observation from the original publication regarding heterogeneous response of the monocytes (**Additional file 5: Supplementary Note 2, Fig. 1C**). Among those 20 genes we identified various transcriptional modules that underpin the heterogeneous response in the monocyte cells. This includes alarmins (S100A8,

S100A9, S100A12), oxidative stress response (MT2A), zinc transport (MT1X, MT1G), pro-inflammatory cytokines (IL1B), multiple chemokines and chemoattractants (CXCL1, CXCL3, CCL8; CXCL5, PPBP; CCL2), apoptosis-associated gene (CTSL).

Overall these findings support the relevance of geneBasis for the discovery of the transient, cell state like, transcriptional changes.

References:

1. Lawlor, N. *et al.* Single Cell Analysis of Blood Mononuclear Cells Stimulated Through Either LPS or Anti-CD3 and Anti-CD28. *Front. Immunol.* 12, 636720 (2021).

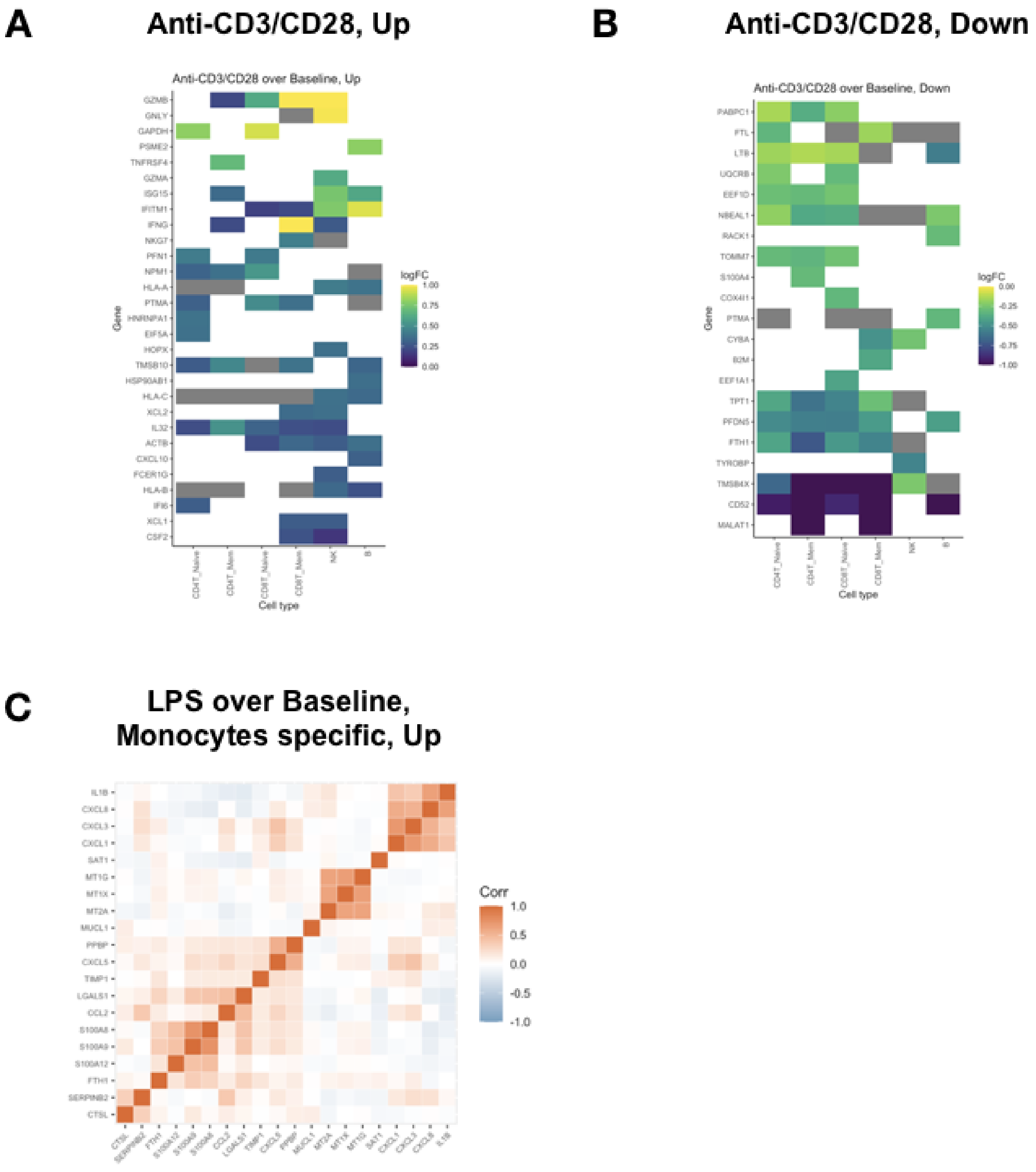

Additional file 5: Supplementary Note 2, Figure 1. A,B) Heatmap plots representing log fold change between anti-CD3/CD28 treated and baseline cells, per cell type (x-axis). Y-axis corresponds to genes detected by geneBasis in at least one cell type. Colour corresponds to log fold change where False Discovery Rate < 0.01 (grey otherwise; additionally, if for a given cell type a given gene was not detected, the corresponding cell will be white). A) corresponds to genes that are up-regulated in anti-CD3/CD28 cells; B) corresponds to genes

that are down-regulated in anti-CD3/CD28 cells. C) Heatmap representing co-expression between genes that are selected by geneBasis and are up-regulated in activated monocytes.

Methods:

Cell state specific selections for activated PBMCs.

To calculate log-normalized counts for the PBMC CITE-seq data, for each batch separately we first calculated size factors for the RNA data using `scran::quickCluster` and `scran::computeSumFactors`, and then calculated log-normalised counts using `batchelor::multiBatchNorm`.

To select cell state specific genes for lymphocyte activation, for each lymphocyte cell type separately, we ran geneBasis for combined baseline and anti-CD3/CD28 cells and calculated the first 25 genes. To select cell state specific genes for monocyte activation, we ran geneBasis for the combined baseline and LPS monocyte cells and calculated the first 25 genes. Prior to gene search, we discarded ribosomal and mitochondrial genes.
