# Supplementary material for: geneBasis: an iterative approach for unsupervised selection of targeted gene panels from scRNA-seq
Source: Genome Biol. 2021 Dec 6;22:333. doi: 10.1186/s13059-021-02548-z (PMC8650258; doi:10.1186/s13059-021-02548-z)
Supplement: Supplementary file 7 — Additional file 7. Review history. [file 13059_2021_2548_MOESM7_ESM.docx]

Review History

**First round of review**

**Reviewer 1**

**Were you able to assess all statistics in the manuscript, including the appropriateness of statistical tests used?**

Yes, and I have assessed the statistics in my report.

**Comments to author:**

This study describes a very useful computational method that takes advantage of k-NN graphs to identify marker genes in single cell data. In addition, they also describe 3 metrics to evaluate gene panels: 1) whether cells can be correctly assigned to their pre-determined labels; 2) how well a cell's neighborhood is preserved with selected genes; 3) correlation of a gene's expression across all cells to its expression across k neighbors in true versus reduced graphs. Overall this is a very sound and useful method and authors have benchmarked it using different datasets and compared to alternative methods: SCMER and scGeneFit. The tool also comes up with very useful features; e.g., evaluating a pre-selected panel or selecting a certain number of genes, ability to handle batch effects. I am very supportive of the publication of this work, I have some minor comments to further improve the presentation.

1) Among the 3 metrics proposed, the first two are very intuitive, third one is more convoluted. Can the authors provide further justification/explanation on why this is a good metric for gene selection maybe with some example scenarios/genes?

2) Sup Fig2A is very interesting and useful. I recommend authors to move this to the main panel. It also shows some very interesting molecules selected by their method in comparison to SCMER, e.g., INS, GCG in pancreas.

3) It would be good to clarify how this method should be used with known batches. There are multiple vignettes provided and it would be great to have one focusing on this aspect as well.

4) It is very intriguing that this method also captures marker genes for cell states. It would be great if the authors can apply this on activated cells to demonstrate this feature. E.g., https://data.humancellatlas.org/explore/projects/efea6426-510a-4b60-9a19-277e52bfa815

Duygu Ucar

**Reviewer 2**

**Were you able to assess all statistics in the manuscript, including the appropriateness of statistical tests used?**

Yes, and I have assessed the statistics in my report.

**Comments to author:**

In this study Missarova et al developed a new algorithm, geneBasis, for selecting small gene panels from single-cell RNA-seq data for targeted analyses, such as RNA in situ mapping. To this end they worked out an iterative strategy to grow a gene set by sequentially adding an informative gene based on a Minkowski distance. They then developed three metrics to assess the performance of a gene panel, and demonstrated that geneBasis performs better than SCMER, in terms of both neighborhood preservation scores and computational speed, using four different data sets from multiple sources. The authors further demonstrated that geneBasis is robust in the present of batch effects or unbalanced cell type composition. Finally, they showed that genes selected by geneBasis not only capture cell-type differences, but also transcriptional differences associated with cell states, the latter are in general more subtle and difficult to detect. Overall, this method represents an advance on the methodology of gene selection. As the field of single-cell analysis is exploding with an exponential increase of scRNA-seq data sets, geneBasis is a very timely tool that might be adopted widely across the community.

While I think geneBasis is addressing a major need in the field, and its performance appears to be very good, I do see a major gap in the method demonstration and benchmarking parts. All the results presented in the manuscript made a convincing case that the gene panels selected by geneBasis can effectively capture the cell types or cell states, in the original data sets from which the genes were selected. However, the real applications of geneBasis, or other algorithms in this category, are to ensure that the gene panels will perform well with the RNA in situ assays, or targeted scRNA-seq for screening purposes. Those experimental methods are more or less different from the methods used for generating the original scRNA-seq data. So in the users' hands, the final data sets will have major technical differences such as dynamic range, molecules detected per cell, or linearity. Exactly how the gene panel will perform with the presence of those technical differences was not characterized in this manuscript at all. This would leave a major uncertainty on the real world performance.

The authors should at least take one in situ data set, such as one of those published seqFISH+ or MERFISH data set, and report the performance metrics, on gene panels of several sizes. As a minor point, some of the supplementary subfigures were not mentioned in the main text.

**Authors Response**

**Point-by-point responses to the reviewers’ comments:**

Reviewer #1:

This study describes a very useful computational method that takes advantage of k-NN graphs to identify marker genes in single cell data. In addition, they also describe 3 metrics to evaluate gene panels: 1) whether cells can be correctly assigned to their pre-determined labels; 2) how well a cell's neighborhood is preserved with selected genes; 3) correlation of a gene's expression across all cells to its expression across k neighbors in true versus reduced graphs.

Overall this is a very sound and useful method and authors have benchmarked it using different datasets and compared to alternative methods: SCMER and scGeneFit. The tool also comes up with very useful features; e.g., evaluating a pre-selected panel or selecting a certain number of genes, ability to handle batch effects. I am very supportive of the publication of this work, I have some minor comments to further improve the presentation.

*We thank the reviewer for such positive comments on our work.*

1) Among the 3 metrics proposed, the first two are very intuitive, third one is more convoluted. Can the authors provide further justification/explanation on why this is a good metric for gene selection maybe with some example scenarios/genes?

*We apologise for the lack of clarity here. As pointed out by the reviewer, cell type and cell based metrics estimate how well we preserve a manifold with any given selection, and therefore provide an intuitive evaluation of whether the selected panel is ‘complete’ or not. In cases where the selected panels are deemed to be incomplete, the third metric - gene prediction score - provides information regarding genes that are specifically missing from the selections. Once they are identified, a user can decide whether the ‘missing genes’ are of interest for inclusion in the final panel.*

*Specifically, the gene prediction score is calculated by taking the ratio between how well a gene’s expression can be ‘explained’ from the Selection graph (i.e. the Spearman correlation across cells between the gene’s actual expression in a cell and its average expression across neighbours in the ‘Selection’ graph or ‘Correlation-Selection’) and how well a gene’s expression can be ‘explained’ in the best case scenario i.e. in the ‘True’ graph (‘Correlation-True’). Using this approach, for any given panel, genes with a low gene prediction score can be regarded as having a transcriptional signal that is not as well preserved in the Selection compared to the True graph, thereby warranting additional exploration. Moreover, if a certain transcriptional program is systematically absent from the current gene panel multiple genes associated with the program will be missing from the library. These transcriptional programmes can be discovered as co-expressed modules containing genes with low gene prediction scores.*

*To further elucidate and describe the intuition behind the gene prediction score, we manually selected a gene panel for the ‘Mouse embryo’ dataset in which we ensured the absence of genes associated with Endothelial cells. Specifically, we selected a panel of 50 genes using geneBasis and manually discarded two genes (Plvap, Lyve1) associated with the “Endothelium” cell type. While Plvap is a strong and homogenous “Endothelium” marker, Lyve1 is expressed in a subset of Endothelium cells and further explains heterogeneity within endothelial cells.*

*For the selected panel of 48 genes, we calculated the gene prediction score for the whole transcriptome (Fig. R1A). We suggest that if a gene cannot be explained well in the True graph then the estimated gene score for this gene is not meaningful. Accordingly, we set a minimal threshold for Correlation-True equal to 0.25 (it can be tuned manually in the relevant functions of the package; Fig. R1A). Among the remaining genes, eleven have a gene score lower than 0.75 (also the default parameter) suggesting that the current panel does not explain their expression profile well. Interestingly, two genes - Lyve1 and Stab2 - show high co-expression (Fig. R1B,C) highlighting that the transcriptional program associated with Lyve1+ endothelial cells is systematically under-explained in the selected library.*

*Overall, we suggest that cell- and gene-based metrics provide a complementary basis to evaluate the quality of the designed gene panel.*

*Figure R1: A) Scatter plot representing the co-distribution of Correlation-True (x-axis) and gene prediction score = Correlation-Selection/Correlation-True (y-axis) for each gene. Genes that cannot be explained well in the True graph (separated by vertical dashed line) are coloured in grey; genes that cannot be explained well in the Selection graph relative to the True graph (separated by horizontal dashed line) are coloured in red. B) Co-expression plot for ‘red’ genes that are poorly explained in the Selection graph compared to the True graph. C) UMAP plots coloured by (from grey (= low) to green (= high)) expression of Lyve1 (left) and Stab2 (right).*

2) Sup Fig2A is very interesting and useful. I recommend authors to move this to the main panel. It also shows some very interesting molecules selected by their method in comparison to SCMER, e.g., INS, GCG in pancreas.

*We thank the reviewer for the positive comment and appreciation of this figure. In the corresponding paragraph we attempt to provide a hypothesis for the discrepancies between the two methods, particularly when a small number of genes is selected. We hypothesize that the strong degree of L1-regularisation (associated with low number of genes selected) used in SCMER does not control for the absence of collinearity between selected genes, which is supported by observing the high degree of co-expression between genes selected by SCMER when compared to geneBasis, especially when the number of genes is low (Supp. Fig. 2A).*

*Nevertheless, it is likely that when the strength of L1-regularisation in SCMER is more relaxed (i.e. more genes can be selected), the overall properties of the genes selected by SCMER might change. Specifically, it is possible that aforementioned genes of interest such as INS and CGC will be selected by SCMER when more genes are allowed (i.e. the model is less regularized). Consequently, whilst we feel it is appropriate to highlight the collinearity of genes selected by SCMER in low complexity libraries, we would not like to assert that SCMER is incapable of highlighting important genes. Subsequently, we feel that this observation is better presented in a supplementary rather than a main text figure.*

*However, we agree with the reviewer that it is worth highlighting the top genes selected by geneBasis in the main figure and have accordingly added new panel 2A (all other panels were renumbered accordingly) representing co-expression of the top 10 genes selected by geneBasis.*

3) It would be good to clarify how this method should be used with known batches. There are multiple vignettes provided and it would be great to have one focusing on this aspect as well.

*Thank you for the suggestion. We now include the section “Batch effect” in vignette #2 (accessible from https://github.com/MarioniLab/geneBasisR and through the direct link: https://rawcdn.githack.com/MarioniLab/geneBasis_tutorials/7aedf70db1fff6f4b616c07a36789eecc097a286/geneBasis_mouseEmbryo_extended.html#4_Batch_effect). In this section we show how to perform gene selection with and without specifying the batch. Additionally, we compare two selections and verify that selection without the batch includes ‘technically variable’ genes that are variable between batches, but do not represent relevant biological variability. We further suggest including the batch (if possible) in order to avoid selection of ‘technically variable’ genes.*

4) It is very intriguing that this method also captures marker genes for cell states. It would be great if the authors can apply this on activated cells to demonstrate this feature. E.g., https://data.humancellatlas.org/explore/projects/efea6426-510a-4b60-9a19-277e52bfa815

*We thank the reviewer for acknowledging this aspect of our approach, and we agree that it is important to demonstrate/benchmark how the algorithm selects cell state genes in various scenarios. As suggested by the reviewer, we applied geneBasis to the aforementioned study (Lawlor et al. 2021). Please find below a paragraph describing the results of this analysis, which we have added as a supplementary note that is appropriately referenced in the main text.*

*To evaluate whether we can detect transcriptional changes upon lymphocyte and monocyte activation we applied geneBasis to the CITE-seq dataset profiling PBMCs from healthy donors that were treated with anti-CD3/CD28 and LPS (Lawlor et al. 2021). Cell types were assigned using marker cell surface proteins, and shifts in transcriptional programs upon lymphocytes and monocytes activation were characterized within individual cell types. Overall, anti-CD3/CD28 treatment activated all lymphocytes, with some genes being ubiquitously altered in their expression whereas others showed cell type specific behaviour. In contrast, LPS treatment specifically altered the transcriptional state of the monocytes, resulting in a heterogeneous response comprised of an early activation and later pro-inflammatory states. To specifically select for genes associated with cell state changes, we performed the panel selections within each cell type separately (Methods).*

*First we analyzed cell type specific cell state changes for the lymphocyte activation dataset (Methods). To do so, we combined baseline cells (no treatment) together with anti-CD3/CD28 cells and applied geneBasis to select the top 25 genes for each lymphocyte related cell type (T cells, B cells and NK). Many of the genes selected in each set of 25 were differentially expressed between baseline and activated cells (Supp. Fig. 8A,B), with some genes being consistently detected across multiple cell types. For example, ACTB, TMSB10 and NPM1 are slightly up-regulated across lymphocytes suggestive of cellular proliferation and movement. On the other hand, some genes were more cell type specific such as a proteasome activity (PSME2) in B cells. Importantly, we identified multiple signals of activation described in the original publication. Among others, we detected interferons and interferon-inducible genes (ISG15, IFNG); chemokines XCL1 and XCL2 in CD8+ cells; cytotoxic molecules GZMB and GNLY in CD8+ and NK cells; and human leukocyte antigens (HLA-B, HLA-C).*

*Next we analyzed monocyte specific cell state changes under LPS treatment. Out of the top 25 genes selected by geneBasis, 20 were up-regulated in activated monocytes, supporting the observation from the original publication regarding heterogeneous response of the monocytes (Supp. Fig. 8C). Among those 20 genes we identified various transcriptional modules that underpin the heterogeneous response in the monocyte cells. This includes alarmins (S100A8, S100A9, S100A12), oxidative stress response (MT2A), zinc transport (MT1X, MT1G), pro-inflammatory cytokines (IL1B), multiple chemokines and chemoattractants (CXCL1, CXCL3, CCL8; CXCL5, PPBP; CCL2), apoptosis-associated gene (CTSL).*

*Overall these findings support the relevance of geneBasis for the discovery of the transient, cell state like, transcriptional changes.*

*Supplementary Notes Figure 2: A,B) Heatmap plots representing log fold change between anti-CD3/CD28 treated and baseline cells, per cell type (x-axis). Y-axis corresponds to genes detected by geneBasis in at least one cell type. Colour corresponds to log fold change where False Discovery Rate < 0.01 (grey otherwise; additionally, if for a given cell type a given gene was not detected, the corresponding cell will be white). A) corresponds to genes that are up-regulated in anti-CD3/CD28 cells; B) corresponds to genes that are down-regulated in anti-CD3/CD28 cells. C) Heatmap representing co-expression between genes that are selected by geneBasis and are up-regulated in activated monocytes.*

Reviewer #2:

In this study Missarova et al developed a new algorithm, geneBasis, for selecting small gene panels from single-cell RNA-seq data for targeted analyses, such as RNA in situ mapping. To this end they worked out an iterative strategy to grow a gene set by sequentially adding an informative gene based on a Minkowski distance. They then developed three metrics to assess the performance of a gene panel, and demonstrated that geneBasis performs better than SCMER, in terms of both neighborhood preservation scores and computational speed, using four different data sets from multiple sources. The authors further demonstrated that geneBasis is robust in the present of batch effects or unbalanced cell type composition. Finally, they showed that genes selected by geneBasis not only capture cell-type differences, but also transcriptional differences associated with cell states, the latter are in general more subtle and difficult to detect. Overall, this method represents an advance on the methodology of gene selection. As the field of single-cell analysis is exploding with an exponential increase of scRNA-seq data sets, geneBasis is a very timely tool that might be adopted widely across the community.

*We thank the reviewer for their positive comments on our work.*

While I think geneBasis is addressing a major need in the field, and its performance appears to be very good, I do see a major gap in the method demonstration and benchmarking parts. All the results presented in the manuscript made a convincing case that the gene panels selected by geneBasis can effectively capture the cell types or cell states, in the original data sets from which the genes were selected. However, the real applications of geneBasis, or other algorithms in this category, are to ensure that the gene panels will perform well with the RNA in situ assays, or targeted scRNA-seq for screening purposes. Those experimental methods are more or less different from the methods used for generating the original scRNA-seq data. So in the users' hands, the final data sets will have major technical differences such as dynamic range, molecules detected per cell, or linearity. Exactly how the gene panel will perform with the presence of those technical differences was not characterized in this manuscript at all. This would leave a major uncertainty on the real world performance. The authors should at least take one in situ data set, such as one of those published seqFISH+ or MERFISH data set, and report the performance metrics, on gene panels of several sizes.

*We want to thank the reviewer for bringing up an important point, and for providing an excellent suggestion on how to address it. Generally speaking, geneBasis is a method to facilitate a gene panel selection for any, either already feasible or yet to be invented, technology that requires a targeted gene panel design. Since by default we use scRNA-seq as the manifold that encodes overall transcriptional variability, an underlying assumption for successful library design via geneBasis is that count matrices of the two data modalities -- scRNA-seq and the assay of interest - show comparable degrees of scaling, variation and overall expression distributions. In other words, the assumption is that genes that selected by geneBasis (from scRNA-seq) will also show sufficient variability and specificity within an assay of interest, thus allowing similar discrimination between cell types and cell states. If, for any given assay, this assumption fundamentally does not hold, it is likely that geneBasis will perform sub-optimally and an alternative library design method is better suited.*

*Practically speaking, at least at the present, we assumed that the main assay for which geneBasis is relevant, are -FISH based spatial transcriptomics techniques. For multiple seqFISH and MERFISH datasets, relevant scRNA-seq datasets have been utilized to select cell type specific markers, and it has been shown that these markers successfully resolve corresponding cell types in situ.*

*To further support that the genes selected by geneBasis successfully resolve annotated cell types in -FISH data, we applied geneBasis to seqFISH+ data for mouse olfactory bulb (Eng et al., 2019). Please find below the paragraph describing the results of this analysis. We added this paragraph to the manuscript, together with Supplementary Figure 8 (the numbering of other Supplementary figures was adjusted accordingly to satisfy the new order of the paragraphs).*

*geneBasis selects genes that resolve cell types in seqFISH+ data.*

*Technical differences between scRNA-seq and FISH-based technologies affect the scaling, magnitude of variance and overall distribution of the expression, either across multiple genes or in a gene specific manner (Asp, Bergenstråhle, and Lundeberg 2020). To assess whether selected by geneBasis gene panel resolves cell types not only in the scRNA-seq from which it was derived, but in the matched -FISH data themselves, we applied geneBasis to a published seqFISH+ study profiling the olfactory bulb of a mouse using a 10000 gene probe set (Eng et al. 2019). To select a ranked gene panel, we used corresponding scRNA-seq from (Zeisel et al. 2018), covering various anatomical units of a mouse brain.*

*First, to ensure that the reference scRNA-seq dataset sufficiently covers the heterogeneity captured in the seqFISH+ sample, we generated a joint embedding in the latent space between scRNA-seq and seqFISH+ cells (Methods, Supp. Fig. 8A). Both data sets contain cluster/cell type labels, and we confirm that cells from the same or comparable cell types fall in the same latent spaces (Supp. Fig. 8A,B). This suggests that biological variability between cells from different cell types outweigh technical differences between scRNA-seq and seqFISH technologies, thereby supporting the notion of using scRNA-seq data to select relevant for seqFISH genes.*

*Next we applied geneBasis to the corresponding scRNA-seq dataset and considered the top 150 genes (across genes probed in seqFISH+ assay) and assessed whether the selected genes can resolve distinct seqFISH+ clusters identified using the full dataset. To do so, we estimated the cell type mapping accuracy for normalized logcounts from seqFISH+ using only the selected panel (Supp. Fig. 8C, left panel). To estimate how much scaling differences between scRNA-seq and seqFISH factor in the performance of the library, we added a positive control where we applied geneBasis to the seqFISH+ data itself, and then evaluated the performance of this panel using the seqFISH+ data (Supp. Fig. 8C, center panel). Finally, to estimate a per cell type upper bound of cell type mapping accuracy, we calculated cell type mapping accuracy on seqFISH+ data using all 10000 genes (Supp. Fig. 8C, right panel). Overall, the selection from seqFISH+ shows a moderate increase in cell type mapping accuracy for some cell types when compared to the selection from scRNA-seq. Nevertheless, both selections show rapid convergence to the estimated upper limit with the exception of a couple rare cell types such as shown in Supp. Fig. 8C,D. Thus, we suggest that geneBasis captures biological heterogeneity that is capable of overcoming technical differences between scRNA-seq and seqFISH.*

*Supplementary Figure 8. geneBasis selects genes that recover biological heterogeneity in seqFISH datasets.*

*A) UMAP plots representing joint embedding for matched scRNA-seq (left) and seqFISH+ (right) of an olfactory bulb of a mouse. Colours correspond to respectively annotated cell types/clusters. B) Heatmap representing confusion matrix for seqFISH+ cells between cluster originally assigned in seqFISH+ (X-axis) and mapped Class from scRNA-seq (Y-axis). C) Heatmaps representing cell type mapping accuracy (for seqFISH+ data) for the selection of 150 genes derived from scRNA-seq (left); selection of 150 genes derived from seqFISH+ (center); all 10000 genes probed in seqFISH+. D) Cell type mapping accuracy as a function of the number of genes in the panel. Each facet corresponds to the cluster from seqFISH+, colours correspond to the origins of the gene panel.*

As a minor point, some of the supplementary subfigures were not mentioned in the main text.

*Thank you for alerting us to this, we have now made corresponding corrections in the main text. Specifically, we added the appropriate mentions for panels Fig. 4I; Supp. Fig. 6A, C; Supp. Fig. 7A, E; Supp. Fig. J,K. Additionally, we added the sentence clarifying panels of Supp. Fig. 6 regarding selections within specific cell types (in the paragraph “geneBasis resolves rare cell types and unannotated inter-celltype variability.”):*

*Additionally, the selection of genes when performed only within Cardiomyocytes also contains genes marking different Me-clusters (Supp. Fig. 6J).*

*References:*

*Asp, Michaela, Joseph Bergenstråhle, and Joakim Lundeberg. 2020. “Spatially Resolved Transcriptomes-next Generation Tools for Tissue Exploration.” BioEssays: News and Reviews in Molecular, Cellular and Developmental Biology 42 (10): e1900221.*

*Eng, Chee-Huat Linus, Michael Lawson, Qian Zhu, Ruben Dries, Noushin Koulena, Yodai Takei, Jina Yun, et al. 2019. “Transcriptome-Scale Super-Resolved Imaging in Tissues by RNA seqFISH.” Nature 568 (7751): 235–39.*

*Lawlor, Nathan, Djamel Nehar-Belaid, Jessica D. S. Grassmann, Marlon Stoeckius, Peter Smibert, Michael L. Stitzel, Virginia Pascual, Jacques Banchereau, Adam Williams, and Duygu Ucar. 2021. “Single Cell Analysis of Blood Mononuclear Cells Stimulated Through Either LPS or Anti-CD3 and Anti-CD28.” Frontiers in Immunology 12 (March): 636720.*

*Zeisel, Amit, Hannah Hochgerner, Peter Lönnerberg, Anna Johnsson, Fatima Memic, Job van der Zwan, Martin Häring, et al. 2018. “Molecular Architecture of the Mouse Nervous System.” Cell 174 (4): 999–1014.e22.*

**Second round of review**

**Reviewer 1**

Authors properly addressed my comments, I think this is a great addition to the field.

**Reviewer 2**

In this revision, the authors have added a new section applying their method to seqFISH data, and demonstrate the performance across different technologies. I have no more concerns, and believe that a timely publication of this manuscript will be beneficial to the community.
